# Cinnamic Acid Bornyl Ester Derivatives from *Valeriana wallichii* Exhibit Antileishmanial *In Vivo* Activity in *Leishmania major*-Infected BALB/c Mice

**DOI:** 10.1371/journal.pone.0142386

**Published:** 2015-11-10

**Authors:** Anita Masic, Ana Maria Valencia Hernandez, Sudipta Hazra, Jan Glaser, Ulrike Holzgrabe, Banasri Hazra, Uta Schurigt

**Affiliations:** 1 Institute for Molecular Infection Biology, University of Wuerzburg, Wuerzburg, Germany; 2 Department of Pharmaceutical Technology, Jadavpur University, Kolkata, India; 3 Institute of Pharmacy and Food Chemistry, University of Wuerzburg, Wuerzburg, Germany; National Center for Cell Science, INDIA

## Abstract

Human leishmaniasis covers a broad spectrum of clinical manifestations ranging from self-healing cutaneous leishmaniasis to severe and lethal visceral leishmaniasis caused among other species by *Leishmania major* or *Leishmania donovani*, respectively. Some drug candidates are in clinical trials to substitute current therapies, which are facing emerging drug-resistance accompanied with serious side effects. Here, two cinnamic acid bornyl ester derivatives (**1** and **2**) were assessed for their antileishmanial activity. Good selectivity and antileishmanial activity of bornyl 3-phenylpropanoate (**2**) *in vitro* prompted the antileishmanial assessment *in vivo*. For this purpose, BALB/c mice were infected with *Leishmania major* promastigotes and treated with three doses of 50 mg/kg/day of compound **2**. The treatment prevented the characteristic swelling at the site of infection and correlated with reduced parasite burden. Transmitted light microscopy and transmission electron microscopy of *Leishmania major* promastigotes revealed that compounds **1** and **2** induce mitochondrial swelling. Subsequent studies on *Leishmania major* promastigotes showed the loss of mitochondrial transmembrane potential (ΔΨ_m_) as a putative mode of action. As the cinnamic acid bornyl ester derivatives **1** and **2** had exhibited antileishmanial activity *in vitro*, and compound **2** in *Leishmania major*-infected BALB/c mice *in vivo*, they can be regarded as possible lead structures for the development of new antileishmanial therapeutic approaches.

## Introduction

Human leishmaniasis is a vector-borne parasitic disease that is caused by more than 20 species of the protozoan genus *Leishmania* [1]. The term “leishmaniasis” covers a variety of clinical manifestations ranging from self-healing to lethal infections. Cutaneous leishmaniasis (CL) causes substantial morbidity with an estimated annual incidence of 800.000 cases, whereas the visceral form of leishmaniasis (VL) is responsible for about 300.000 new infections per year with a total mortality rate of 30.000 [2]. Currently, the few clinically approved drugs against human leishmaniasis are becoming more unreliable due to severe side effects [3] and the appearance of drug resistance problems [4, 5]. Therefore, there is an urgent need for new therapeutic approaches against the most common forms of leishmaniasis.

Following the identification of antileishmanial lead compounds by means of *in vitro* assays, well established animal models are usually chosen to investigate the potential of drug candidates *in vivo*. BALB/c mice have been extensively used, mainly due to their susceptibility to a variety of *Leishmania* species, and particularly for *Leishmania* (*L*.*) major* infection studies [6, 7].

For the last few years, many naturally occurring compound classes such as chalcones and quinolines were found to have antileishmanial activity *in vitro* and in experimental mouse models [8, 9]. Recently, a semi-pure fraction of a chloroform extract of *Valeriana* (*V*.) *wallichii* with significant antileishmanial activities and with IC_50_ values lower than 10 μg/mL in addition to an apparent apoptosis-like cell death induction were identified [10]. Further fractionation resulted in caffeic acid bornyl ester exhibiting good antileishmanial activity against *L*. *major* promastigotes [11]. This finding prompted the synthesis of a library of related compounds in order to establish structure-activity relationships (SAR). In general, compounds having a brenzcatechin moiety were found to be active, but also cytotoxic against host cells due to oxidation and formation of a highly reactive Michael system, whereas analogue compounds without the 3,4-dihydroxy substitution at the phenyl ring (e.g. compound **1**) were still active but less toxic [12]. Furthermore, the typical Michael system of the caffeic acid moiety could be omitted to obtain an active but non-toxic phenylpropanoic acid ester **2** [12].

The present study aims to determine the effect of compound **2** treatment on CL *in vivo* and to elucidate the cell death mechanism induced by both the compounds (**1** and **2**) in *L*. *major* parasites. Our results suggest that the approach of using bornyl caffeate isolated from *V*. *wallichii* and related derivatives is efficient to identify and optimize antileishmanial lead structures showing *in vitro* and *in vivo* activity towards *L*. *major* in BALB/c mice.

## Material and Methods

### Synthesis of compounds 1 and 2

(-)-Bornyl cinnamate (**1**) and (-)-bornyl 3-phenylpropionate (**2**) were synthesized as described previously [12].

### *Leishmania* strains and cultivation methods

The cloned virulent *L*. *major* parasite (strain: MHOM/IL/81/FE/BNI) was maintained by continuous passage in female BALB/c mice (Government of Lower Franconia, Germany, permission number: 55.2-2531.01-26/12). *L*. *major* amastigotes were isolated from lesions as described previously [13] and promastigotes were grown *in vitro* in blood-agar cultures at 27°C, 5% CO_2_, and 95% humidity.

Luciferase-transgenic (Luc-tg) *L*. *major* has been generated as described previously [14]. The virulence of Luc-tg *L*. *major* was maintained by passage in female BALB/c mice and promastigotes were grown *in vitro* in blood agar cultures with addition of 50 μg/mL hygromycin B using the same conditions as described above.

*L*. *donovani* promastigotes (strain: MHOM/IN/1983/AG83) were obtained from the Indian Institute of Chemical Biology, Kolkata, India. Promastigotes were cultured in Schneider’s insect medium (Sigma-Aldrich Co., St. Louis, USA) as described elsewhere [15].

### *In vitro* antileishmanial activity against *Leishmania* parasites and cytotoxicity studies

Antiparasitic activities of compounds **1** and **2** dissolved in dimethyl sulfoxide (DMSO, E. Merck, India) against *L*. *donovani* (AG83) promastigotes were determined by a quantitative colorimetric assay using the 3-(4, 5- dimethylthiazol-2-yl)-2, 5-diphenyl tetrazolium bromide (MTT) (Sisco Research Laboratory, Mumbai, India) test as described elsewhere [16]. The compounds were tested at concentrations of 10 μM, 50 μM, and 100 μM and IC_50_ values (concentration of compound, which inhibited at least 50% cell metabolic activity) for each compound were determined from respective dose–response curves using Origin 5.0 software (Microcal Software, Inc., Northampton, MA, USA).

The activity of the two tested compounds **1** and **2** against *L*. *major* parasites was evaluated using the colorimetric AlamarBlue assay as described previously [17]. The compounds were tested at increasing concentrations ranging from 0.1 μM—100 μM for 24 h. AlamarBlue was added for additional 48 h and the optical density was measured with Multiskan Ascent enzyme-linked immunosorbent assay (ELISA) reader (Thermo Electron Corporation, Dreieich, Germany) using a test wavelength of 540 nm and a reference wavelength of 630 nm. Absorbance in the absence of compounds was set as 100% parasite growth.

A detailed protocol of antileishmanial investigations against intracellular *L*. *major* amastigotes residing within bone marrow-derived macrophages (BMDM) is described elsewhere [14]. Briefly, 2 × 10^5^/ml BMDM were infected with Luc-tg *L*. *major* promastigotes at a ratio of 1:15 for 24 h. These infected BMDM were cultured in the absence or presence of increasing concentrations of the tested compounds **1** and **2** for 24 h prior to the supplement of Britelite plus (PerkinElmer, Waltham, MA, USA) for 5 min. Britelite plus is a very sensitive assay for the quantification of firefly luciferase in *Leishmania* parasites [14]. The luminescence was measured using a Victor X Light 2030 luminometer (PerkinElmer, Waltham, MA, USA).

BMDM were generated in complete RPMI medium as described previously [14]. On day six, the cytotoxicity against 2 × 10^5^ cells/ml was tested at increasing concentrations of compounds **1** and **2** for 48 h. Human embryonic kidney HEK 293 T cells (ATCC, Wesel, Germany) were cultured in Dulbecco´s Modified Eagle Medium (DMEM) (Life Technologies, Darmstadt, Germany), with 10% FCS (PAA Laboratories, Germany). According to the standard operating procedures (SOP) within the Collaborative Research Center 630 (SFB 630) 2 × 10^4^ HEK 293 T cells/ml were tested at increasing concentrations of compounds **1** and **2** for 48 h. Human liver carcinoma HepG2 cells (ATCC) were cultured in RPMI medium (Gibco) with 10% FCS. 1 × 10^5^ cells/ml HepG2 were tested at increasing concentrations of **1** and **2** for 72 h according to the SOPs. The AlamarBlue assay was performed as described above for *L*. *major* parasites.

### Ethics statement

All *L*. *major* animal experiments were designed and performed in strict accordance with the German Animal Welfare Act (TierSchG) according to the experimental guidelines and procedures approved by the government of Lower Franconia, Germany (permission number: 55.2-2531.01-25/13). The *L*. *major* parasite (strain: MHOM/IL/81/FE/BNI) was originally isolated from an Israeli patient with oriental sore in 1981 and was received from the Bernhard-Nocht-Institute, Hamburg (Germany) [18].

The *L*. *donovani* parasite (strain: MHOM/IN/1983/AG83) was originally isolated from a North Indian VL patient unresponsive to treatment with sodium stibogluconate and was received as a generous gift from Prof. Shyam Sundar, Institute of Medical Sciences, Banaras Hindu University, India [19].

### Mice

Naïve female inbred BALB/c mice weighing 16–18 g were purchased (Charles River Breeding Laboratory, Sulzfeld, Germany), housed in groups of four and were given five days to acclimate prior to treatment. At the onset of the *L*. *major* infection studies the mice were 6–8 weeks of age. All mice were kept under specific pathogen-free conditions in individually ventilated cages (IVC). Environmental conditions were a temperature of 21°C ±2°, humidity of 50% ±10%, lighting of 60 lux and a 12:12 light: dark cycle with lights on at 7 a.m. and off at 7 p.m. Animals were housed in 391×199×160 mm cages (Techniplast GmbH, Hohenpeißenberg, Germany; Seal Safe PLUS cages X-TEMP PPSU) and given access to mouse maintenance food (ssniff Spezialdiaeten GmbH, Soest, Germany; R/M-H maintenance food) and water *ad libitum*. Environmental enrichment included bedding (Abedd LAB and VET Service GmbH, Vienna, Austria; Espe-classic— H0234-40), and one handful of paper tissue nesting material. During housing, animals were monitored daily for health status. No adverse events were observed.

### *In vivo* antileishmanial activity against *L*. *major*

Female BALB/c mice (11 per group; three groups in total) were infected with 2 × 10^5^ *L*. *major* promastigotes in 30 μL phosphate-buffered saline (PBS) into the right hind footpad under isoflurane inhalation anaesthesia using the UniVet Porta system (Groppler, Deggendorf, Germany) [7]. The clinical manifestation of *L*. *major* infection was monitored twice a week by determination of the body weight (g) and footpad size (mm) of the infected (iFP) and the non-infected footpad (niFP) serving as negative control. The footpad swelling describes the difference in mm between the iFP and niFP of individual mice. Three weeks post infection (p.i.) the mice were randomly divided into three groups of 11 for different treatment regimens. On day 21, 25, and 28 p.i. the respective groups were either left untreated, treated intraperitoneally (i.p.) with 100 μl of solvent (infected, 50% DMSO/PBS) or 100 μl 50 mg/kg/day of compound **2** in 50% DMSO/PBS. This high dose of compound **2** was chosen to identify its antileishmanial *in vivo* efficacy in *L*. *major*-infected BALB/c mice. The close monitoring of body weight and footpad swelling was continued until the end of experiment. On day 35 p.i. mice were sacrificed by CO_2_ inhalation and single cell suspensions from the iFP, draining popliteal lymph nodes (pLN) and spleens were obtained. The parasite burden was determined by limiting dilution assays as described previously [20]. It was not necessary to apply any analgesics or anaesthetics during the animal trials. Compound **2** did not have any strong side effects.

### Cultivation conditions for compound 1- and compound 2-induced phenotypic changes in *L*. *major* promastigotes

*L*. *major* parasites harvested from blood agar cultures were washed twice with Dulbecco’s phosphate-buffered saline (DPBS; Invitrogen, Darmstadt, Germany) and centrifuged at 3.000 × g for 10 min. The pellet was suspended in RPMI 1640 medium at a final concentration of 1 × 10^8^ cells/ml. Cells were treated with 1% DMSO, 122.7 μM miltefosine (1-hexadecylphorylcholine; apoptosis inducer), or 100 μM of the tested compounds **1** and **2** in a final volume of 200 μl in 96 well plates. Samples were incubated at 27°C in 5% CO_2_ for several time points between 30 min and 24 h. Transmitted light microscopic, transmission electron microscopic (TEM), and flow cytometric techniques were used to find phenotypic patterns caused by the treatment with the tested compounds.

### Diff-Quik staining for transmitted light microscopy

After incubation for 30 min, 1 h, 2 h, 4 h, and 24 h in the presence of the tested compounds **1** and **2,** promastigotes were harvested and centrifuged in a Cytospin 3 centrifuge (Shandon, Frankfurt, Germany) on microscope slides at 253 × g for 5 min at room temperature (RT). Cytospin preparations of cells were stained using the Differential Quick Stain (Diff-Quik) dye (Medion Diagnostics AG, Duedingen, Switzerland), according to the manufacturers’ protocol with some modifications. Diff-Quik stains the leishmanial nuclei and the kinetoplasts dark purple and the cytoplasm light purple allowing the observation of phenotypic changes within the parasite. Briefly, air-dried cells were fixed by dipping the slides 5 times for 3 seconds in fixative solution. Subsequently the slides were stained with Solution I following Solution II (from Diff-Quik kit) and air-dried between each staining steps. Finally, the samples were rapidly dipped once in ultra-pure water (produced by a TKA purification system apparatus, Niederelbert, Germany) to remove excessive staining solutions. The air-dried cells were analyzed by transmitted light microscopy under a 50× objective on a Nikon ECLIPSE 50*i* microscope equipped with a digital camera (Nikon, Tokyo, Japan). The images were processed using NIS Elements D software (Nikon).

### Transmission electron microscopy (TEM)

*L*. *major* promastigotes were incubated for 2 h in the presence of DMSO, 100 μM of compound **1** and compound **2**, respectively. After the incubation period, the parasites were harvested, contrasted and embedded following the procedure described previously [21]. Ultrathin sections were mounted on 300-mesh grids, stained with uranyl acetate and lead citrate, and analyzed with an EM 10 transmission electron microscope (Carl Zeiss AG, Oberkochen, Germany). To describe morphological changes in a statistical manner, six photographs with approximately 50 cells were taken and the percentage of cells with swollen mitochondria per treatment was determined.

### MitoTracker staining of *L*. *major* promastigotes

*L*. *major* promastigotes were harvested from blood-agar cultures and washed twice with DPBS (900 × g for 5 min at RT). 2 × 10^6^ promastigotes were dissolved in 180 μl RPMI complete medium and seeded in a 96 well plate. Parasites were incubated with 20 μL of compound **1** (final concentration: 100 μM), compound **2** (final concentration: 100 μM) and with 20 μL of DMSO, respectively, for 30 min, 45 min, 60 min, or 120 min at 27°C in a 5% CO_2_. After incubation 2 μl of MitoTracker Red CMXRos (final concentration: 100 mM) (Life Technologies, Darmstadt, Germany) was added for 15 min at 27°C. Samples were directly acquired using a MACSQuant Analyzer (Miltenyi Biotech, Bergisch Gladbach, Germany) and MitoTracker-positive parasites were analyzed using MACS Quant Analyzing software.

### Determination of the cell death phenotype by flow cytometric analysis

The loss of membrane integrity and translocation of phosphatidylserine (PS) to the outside of the cellular membrane can be determined by annexin V (AV) and propidium iodide (PI) staining. Double staining with AV-fluorescein isothiocyanate (FITC) and PI during flow cytometry analysis leads to discrimination between four populations: double negatives or alive cells (AV^-^/PI^-^), double positive or late necrotic/late apoptotic cells (AV^+^/PI^+^), PI-positive and AV-negative or early necrotic cells (AV^-^/PI^+^), and PI-negative and AV-positive or early apoptotic cells (AV^+^/PI^-^) [22, 23].

Parasites were harvested after incubation for 6 h, 10 h, and 24 h in the presence of compounds **1**, **2** and miltefosine (Cayman Chemical Company, MI, USA), respectively, the latter serving as positive control for the induction of apoptotic cell death. DMSO was used as solvent control. Cell staining was performed using an AV-FITC Apoptosis detection kit (Sigma-Aldrich, Saint Louis, MO, USA). Cells were harvested, washed and suspended in 500 μL of binding buffer, 5 μL of AV-FITC and 10 μl of PI. The cells were incubated for 10 min in the dark at RT. Finally, stained samples were immediately analyzed by flow cytometry using MACSQuant analyzer or a flow cytometer (Becton Dickinson, San José, CA, USA).

### Statistical analyses

Data were analyzed using the GraphPad Prism 5 (San Diego, USA) software and values are given as mean ± standard deviation (SD). Footpad swelling of *Leishmania*-infected mice as well as the parasite burden of infected tissues is displayed per individual mouse with 11 mice per group. The unpaired *t*-test was used to compare two independent groups. Differences were considered significant at **, p<0.005.

## Results

### Compounds 1 and 2 revealed antileishmanial activity against *L*. *major* and *L*. *donovani*

**Fig 1:**
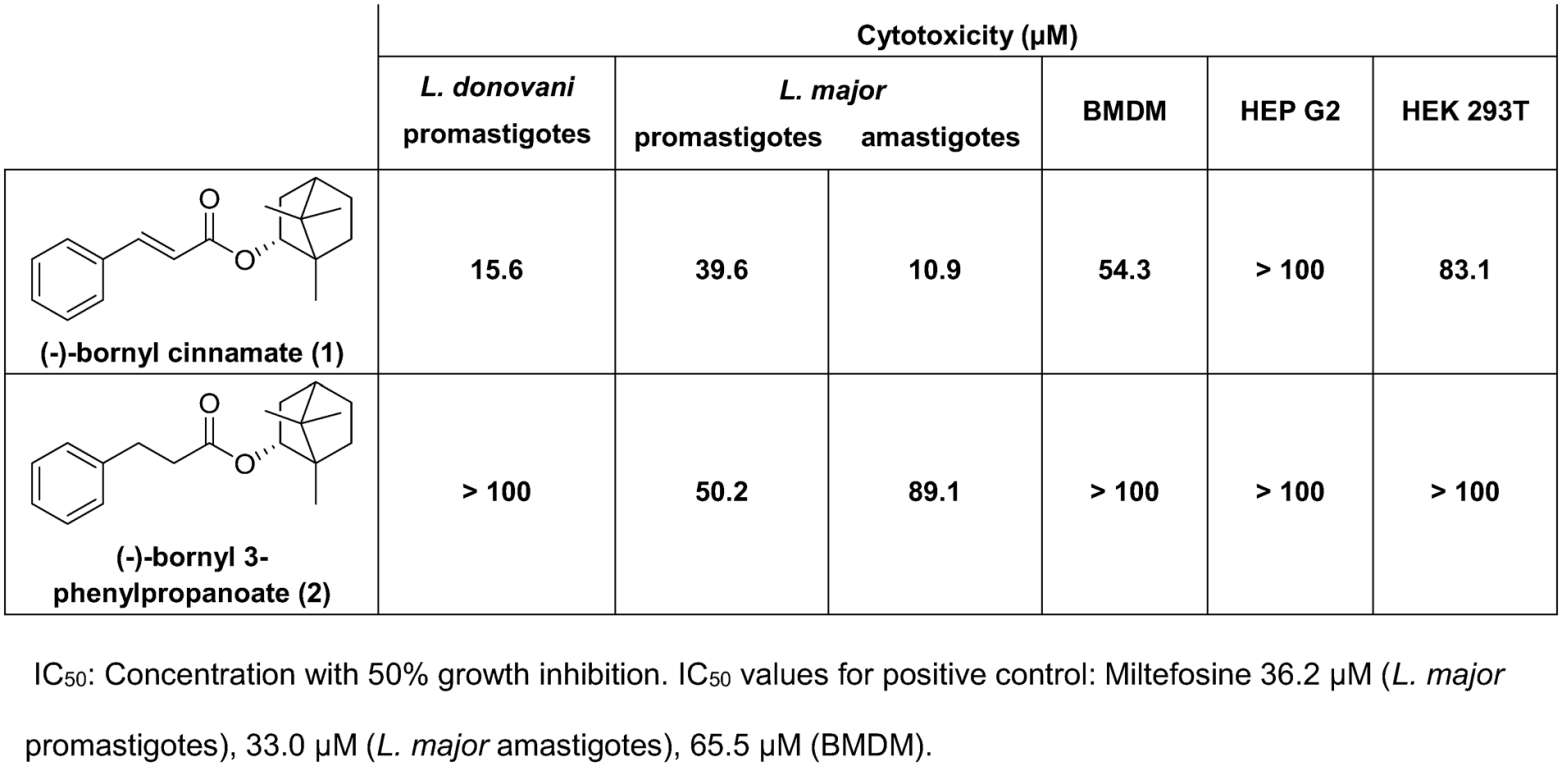
Determination of cytotoxicity and IC_50_ values against *Leishmania* parasites.

For the initial evaluation of the antileishmanial activity of compounds **1** and **2**, a MTT reduction and an AlamarBlue assay with *L*. *donovani* and *L*. *major* parasites were employed, respectively. The cytotoxicity effects of the compounds were investigated in BMDM, HEP G2, and HEK 293T cells (Fig 1). Compound **1** showed the best activity against *L*. *donovani* promastigotes as well as *L*. *major* amastigotes and promastigotes (Fig 1). The half maximal inhibitory concentrations (IC_50_) for compound **1** were between 39.6 μM against *L*. *major* promastigotes and 10.9 μM against *L*. *major* amastigotes, being in the range of miltefosine used as a positive control with an IC_50_ value of 36.2 μM against promastigotes and 33 μM against amastigotes [12]. The cytotoxicity was variable for the three tested cells up to the highest dose of 100 μM.

50% of growth inhibition was not evident after treatment of *L*. *donovani* promastigotes with the bornyl 3-phenylpropanoate (compound **2**) with a dose up to 100 μM, but after treatment of *L*. *major* promastigotes and amastigotes at 50.2 μM and 89.1 μM, respectively.

Interestingly, no cytotoxicity effects against host cell lines were found for the three cell types up to 100 μM, whereas miltefosine displayed an IC_50_ value of 65.5 μM against BMDM.

The determination of IC_50_ values showed strain-specific differences. The cytotoxicity against host cells in compound **1**-treated cells was reduced after omitting the Michael system from this compound resulting in compound **2**, with a moderate activity against *L*. *major* promastigotes and amastigotes.

### Compound 2 exhibited antileishmanial activity against *L*. *major* infection in BALB/c mice

**Fig 2:**
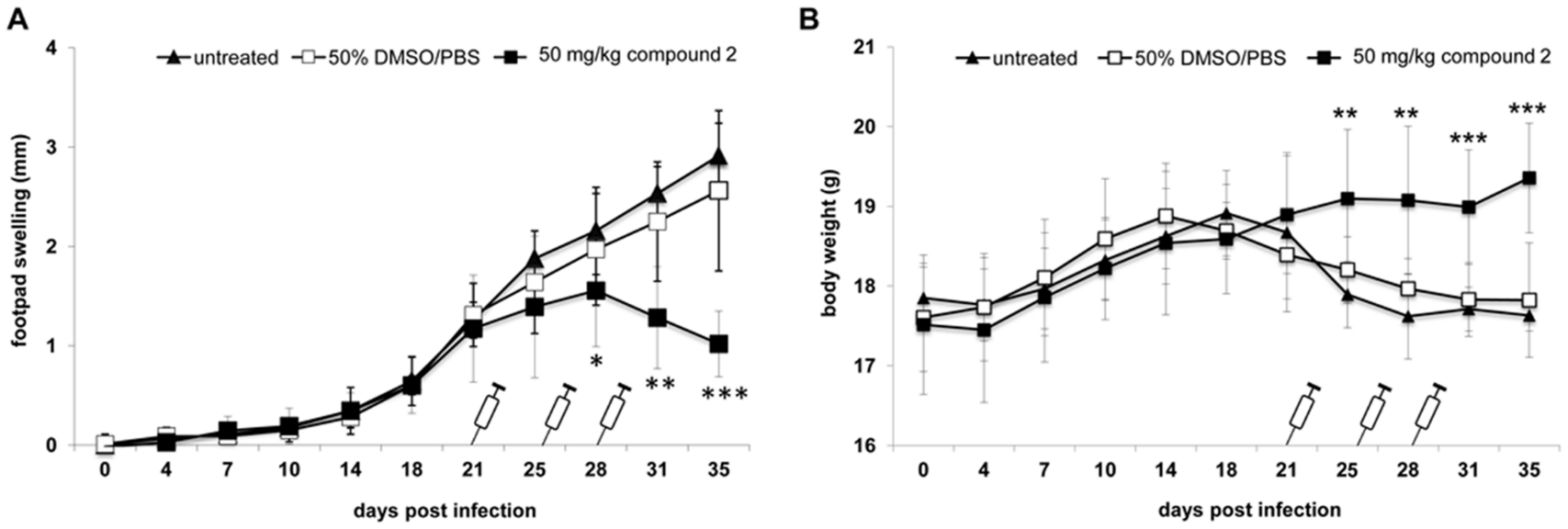
Compound 2 treatment mediates recovery from *L*. *major-*induced disease progression in BALB/c mice. Female BALB/c mice (total: n = 33) were infected with 5 × 10^6^ *L*. *major* promastigotes into the right hind footpad. On d 21 post infection BALB/c mice were either treated intraperitoneally with 50 mg/kg compound 2 (■) (n = 11), 50% DMSO/PBS (□) (n = 11) or left untreated (▲) (n = 11). Individual treatment was repeated on d 25 and d 28 post infection. A. Footpad swelling (difference between infected and non-infected footpad in mm) and B. body weight (g) was determined twice a week to monitor clinical manifestation of CL. n = number of animals.

Since compound **2** exhibited no detectable cytotoxicity against the tested mammalian cell types but showed antileishmanial activity against *L*. *major* parasites only (Fig 1), the effect of this compound on *L*. *major*-infected BALB/c mice was investigated. As the translation from *in vitro* findings to *in vivo* efficacy is the bottleneck in successful drug research, an experimental *Leishmania*-infection model was chosen to provide evidence for their antileishmanial activity *in vivo* [24]. Severe swelling and lesion development at the site of infection with ascending parasite dissemination into organs during the onset of infection characterizes this CL infection model [25]. An established CL infection in non-healing BALB/c mice can be observed from week three post infection and is characterized by significant footpad swelling and elevated parasite burden [7]. To investigate the antileishmanial effect in an already established *L*. *major* infection, compound **2** was assessed for its activity in *L*. *major-*infected female BALB/c mice (Fig 2). Consequently, the treatment regimen was started on day 21 with repetitions on days 25 and 28 p.i. to evaluate the efficacy of a short-term treatment regimen [26].

*L*. *major*-infected BALB/c mice treated i.p. with 50 mg/kg/day of compound **2** on days 21, 25, and 28 showed reduced footpad swelling compared to untreated or solvent control-treated mice (Fig 2A). Significant reduction in footpad swelling was already achieved after the second administration of compound **2** on day 25 compared with the footpad swelling of untreated or solvent-treated mice. The triple administration of compound **2** resulted in a reduction of the foot pad swelling, indicating a recovering phenotype upon 28 days post infection. In contrast, an ongoing infection as observed in untreated or solvent-treated mice was accompanied with an increasing footpad swelling. Infected but untreated BALB/c mice reflected the manifestations of experimental CL, characterized by footpad swelling of ~3 mm difference between infected and non-infected footpad (Fig 2A) and significant loss of body weight (Fig 2B) after five weeks post infection. Mice treated with the solvent control showed also clinical manifestations with footpad swelling and loss of body weight, similar to untreated mice. The clinical manifestation and progression of leishmaniasis in the two control groups was also associated with loss of body weight, whereas the body weight of infected mice treated with compound **2** was not affected during the treatment regimen (Fig 2B).

**Fig 3:**
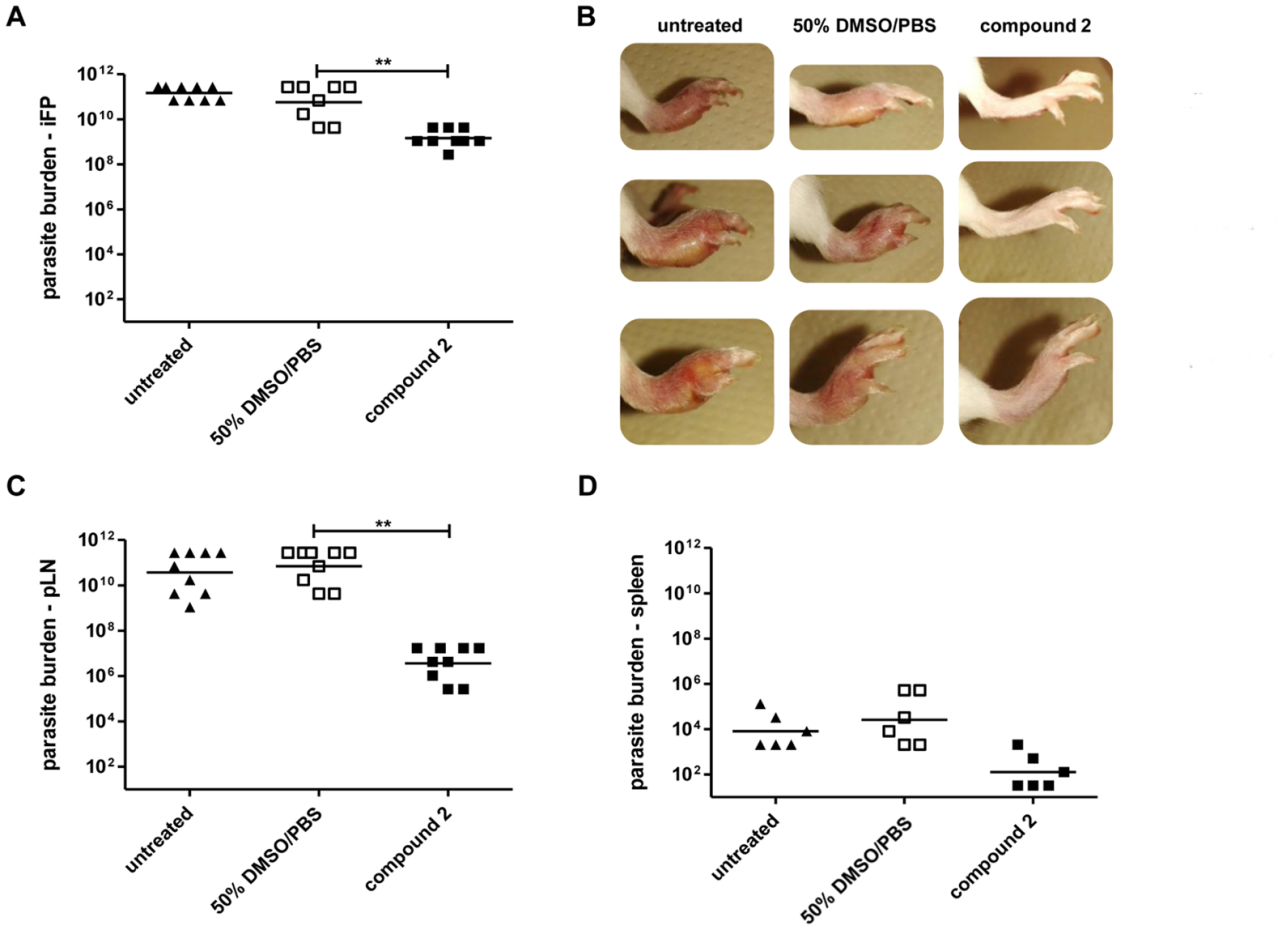
Compound 2 treatment of *L*. *major*-infected BALB/c mice is associated with reduced parasite burden in infected tissues and organs. Female BALB/c mice (n = 33) were infected with 5 × 10^6^ *L*. *major* promastigotes into the right hind footpad. Treatment regimen started on d 21 post infection either using 50% DMSO/PBS (□) (n = 11), 50 mg/kg compound 2 (■) (n = 11) or mice were left untreated (▲) (n = 11). A. At the end of experiment on d 35 all mice (n = 33) were sacrificed and the parasite burden of the infected footpads (iFP) (n = 9) was determined using Limited Dilution Assays (LDA). B. Photographic documentation of the infected footpads of three exemplary mice per group shall reveal the severity of disease progression in non-healing mice and the control of clinical manifestation in compound 2-treated BALB/c mice. C. The parasite burden of the infection site-draining popliteal lymph nodes (pLN) of individual mice (n = 9) or D. spleens of individual mice (n = 6) was determined using LDA. n = number of animals; ** p<0.005; differences in parasite load between 50% DMSO/PBS-treated and compound 2-treated animals did not reach statistical significance for D.

Progressing *Leishmania* infection in BALB/c mice is associated with elevated parasite burdens within infected tissues and organs, whereas a recovering phenotype is associated with reduced parasite levels. Limiting dilutions assays (LDA) confirmed high parasite burden in infected footpads (Fig 3A), at the site of infection-draining popliteal lymph nodes (Fig 3C), and spleens (Fig 3D) of diseased mice. The recovering phenotype in compound **2**-treated BALB/c mice correlated with a ~100 fold reduced parasite burden within the infected footpads, a ~10.000 fold reduced parasite burden within the popliteal lymph nodes (Fig 3C). The parasite burden in the spleen was only numerically reduced, due to a low sample number (Fig 3D). The photographic documentation of three exemplary infected footpads per group demonstrated the clear difference between a compound **2**-treated recovering phenotype and the diseased footpads of control mice at the end of the experiment five weeks post infection (Fig 3B).

Taken together, the short-term treatment of *L*. *major*-infected BALB/c mice with compound **2** was successful in terms of the observed recovery of footpad swelling and parasite burdens compared to untreated mice (Figs 2 and 3).

### Rapid changes in cell morphology and induction of mitochondrial swelling were mediated by compounds 1 and 2 in *L*. *major* promastigotes

**Fig 4:**
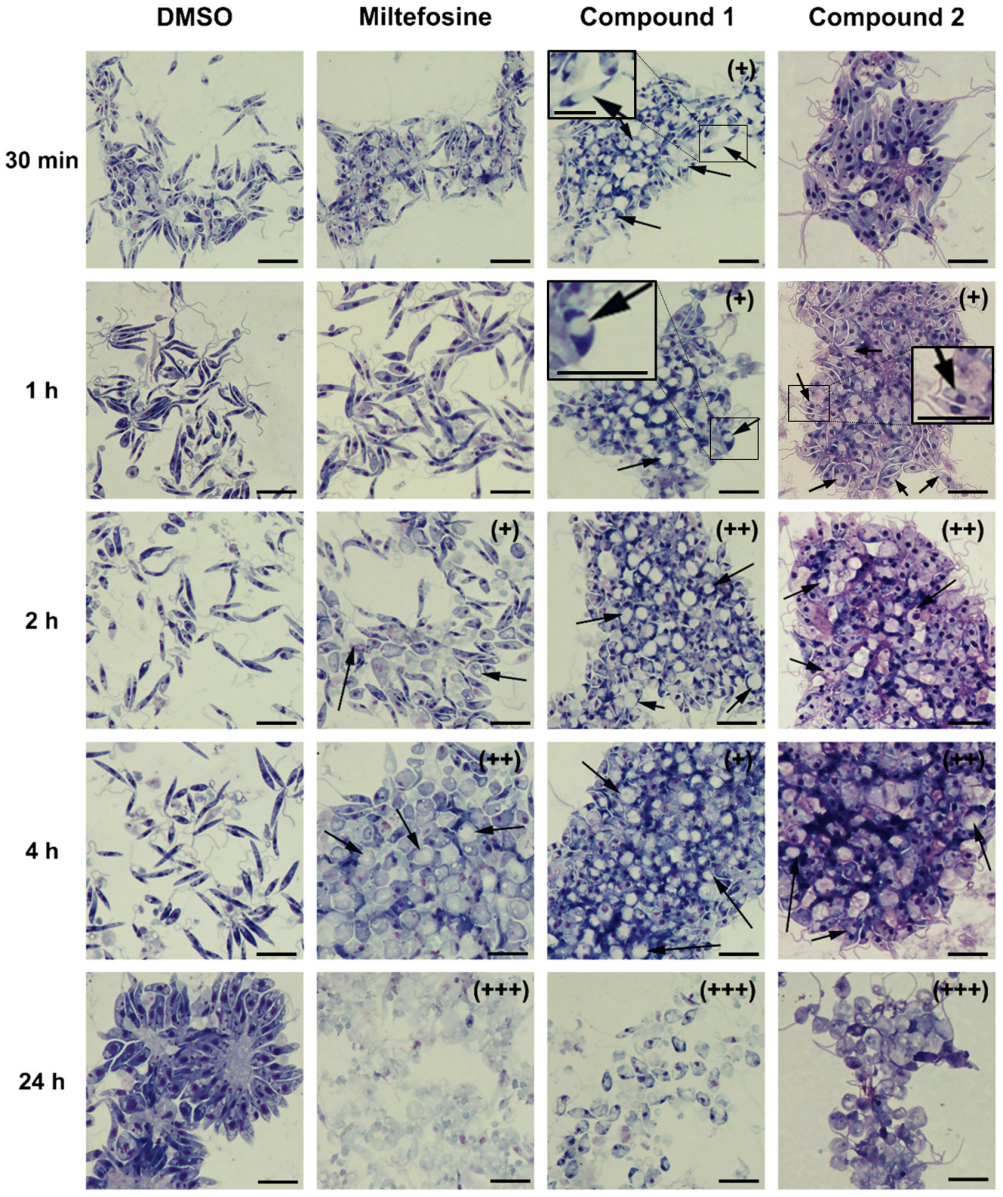
Changes in cell morphology in *L*. *major* promastigotes upon treatment. Compound 1 and 2 induce parasite swelling and vacuolization of *L*. *major* promastigotes at very early time points. *L*. *major* promastigotes were treated with solvent control (1% DMSO), miltefosine (122.7 μM), compound 1 (100 μM), and 2 (100 μM) for 30 min, 1 h, 2 h, 4 h, and 24 h. Cytospin preparations of the cells were stained with Diff-Quik dye and analyzed by transmitted light microscopy under 50× objective. “Plus” symbol (+) was used to represent changes in morphology; two (++) and three (+++) symbols were used as a reference for the severity of the phenotype induced by the different tested compounds and the black arrows to indicate the presence of vacuole-like structures. Black bar indicates 20 μm.

Since IC_50_ values and cytotoxicity assays for compounds **1** and additionally *in vivo* efficiency for compound **2** revealed antileishmanial activity and relatively good selectivity, the effect of these two compounds against *L*. *major* promastigotes was further investigated. First, potential changes of the cell morphology induced by the compounds **1** and **2** were studied by means of transmitted light microscopy. The visual examination of promastigotes incubated with compounds **1** and **2** revealed that the early rounding of cell shape during the first hour of incubation became more prominent over time when compared to control cells (Fig 4). Beside changes in the cell shape of compound-treated promastigotes, the formation of intracellular vacuole-like structures has also been observed within the first hour leading to larger aggregates of cells in vacuolization processes compared to control cells (intracellular vacuoles are better shown in insets of Fig 4). After treatment with miltefosine, compound **1** and **2** for 24 h rounding of shape and shrinkage of almost all cells was observed. No morphological changes were evident in the control cells along the 24 h incubation period (Fig 4). Whereas the apoptosis inducer miltefosine promoted morphological changes and cell disruption after 2 h of treatment, compounds **1** and **2** induced vacuolization and changes in cell morphology. This phenotype was observable in compound **1**-treated promastigotes just after 30 min and in compound **2**-treated promastigotes after 1 h. The antileishmanial effect of compound **1** and **2** as seen by *in vitro* and *in vivo* studies together with the transmission light microscopic investigations identified the potential mode of action against *L*. *major* parasites as a result of changes in the cell morphology and formation of vacuole-like structures within the parasite.

**Fig 5:**
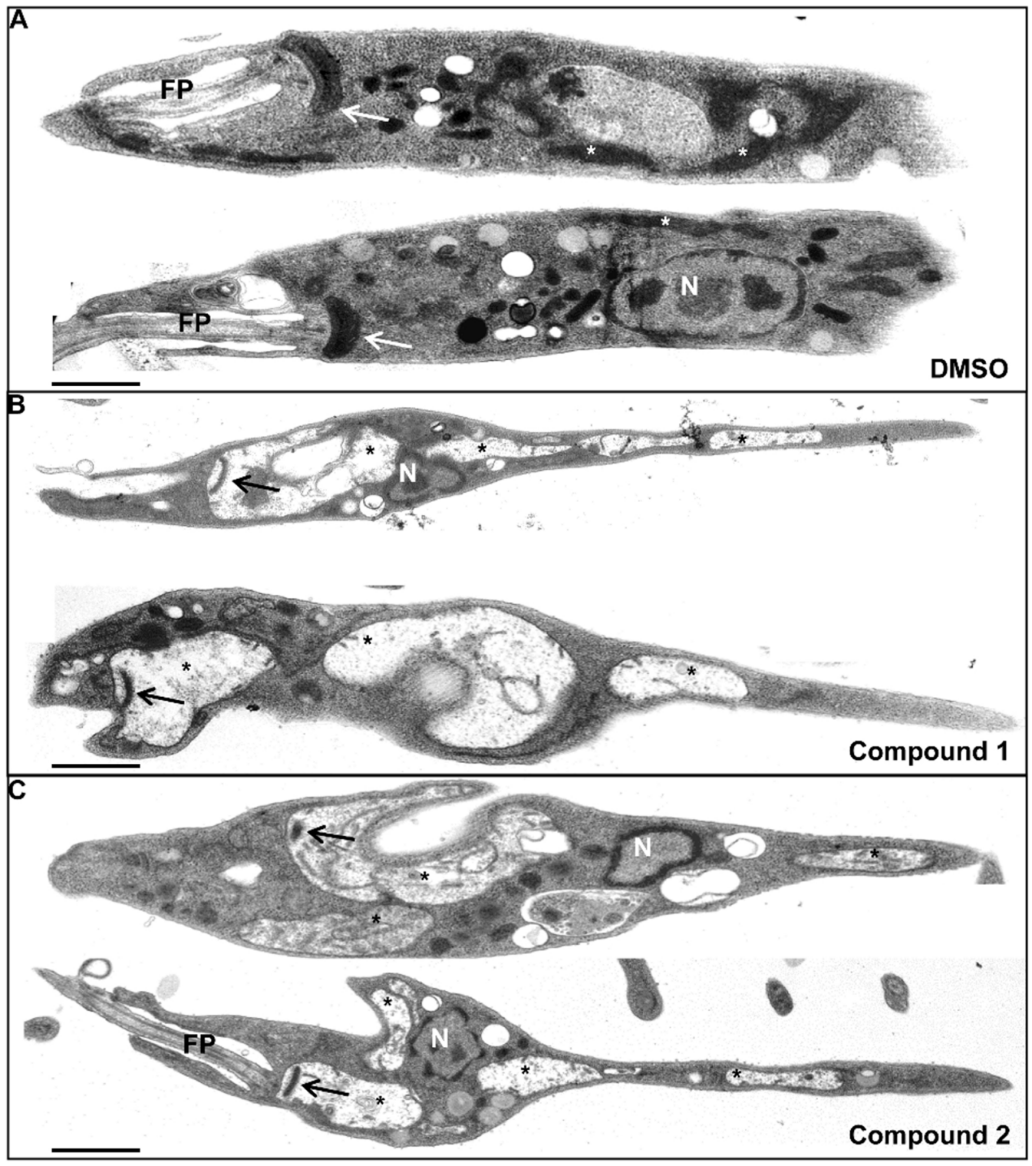
Treatment with compound 1 or 2 induces mitochondrial swelling in *L*. *major* promastigotes. Ultrastructural alterations of mitochondria in compound 1- and compound 2-treated *L*. *major* promastigotes were detected by TEM. A. *L*. *major* promastigotes were incubated for 2 h with DMSO-containing RPMI medium and served as solvent control. B and C show TEM pictures of *L*. *major* promastigotes treated for 2 h with 100 μM compound 1 or 100 μM compound 2, respectively. Two TEM pictures of characteristic parasites are shown per sample. FP, flagellar pocket; N, nucleus; arrow, kinetoplast; white asterisk, mitochondrion; black asterisk, swollen mitochondria. Black bar indicates 1 μm.

The observation of prominent vacuoles and swelling of parasites prompted the elucidation of the origin of those vacuoles by investigating the ultrastructure using TEM. Compared to DMSO-treated promastigotes showing typical morphological characteristics (Fig 5A), parasites treated with compound **1** or **2** displayed large intracellular vacuoles (Fig 5B and 5C) as seen in the light microscopic pictures above (Fig 4). *Leishmania* belongs to the class of *Kinetoplastida*, which are characterized by the presence of a DNA-containing kinetoplast within the giant single mitochondrion being usually localized near the flagellar pocket of the parasite [27]. Considering this, it can be postulated that compound **1** and **2** induced alterations in the ultrastructure of the mitochondria in *L*. *major* promastigotes, as the kinetoplast DNA was clearly visible within the observed vacuoles (Fig 5B and 5C). A difference in percentage of swollen mitochondria in compound- or DMSO-treated parasites was noted. None of the DMSO-treated parasites displayed swollen mitochondria, whereas compound **1** and **2** induced mitochondrial swelling in 39% or 36% of counted parasites after 2 h, respectively. Additionally, compound **1**- and **2**-treated promastigotes (Fig 5B and 5C) displayed a slimmer cell body compared to the DMSO-treated control parasites (Fig 5A). The origin of this phenotype is unclear and must be investigated in further studies.

So far, the leishmanicidal mode of action of the compounds **1** and **2** is most probably due to alterations of the mitochondrial ultrastructure through marked swelling and loss of matrix content, resulting in cell death of the *L*. *major* parasite. Other organelles within the parasite seemed not to be affected by these two compounds, as i.e. the nucleus and other contrasted organelles showed typical morphology.

### Antileishmanial activity of compounds 1 and 2 was attributed to changes in the mitochondrial transmembrane potential of *L*. *major*

The mitochondrial transmembrane potential was investigated as compound-induced mitochondrial dysfunction would lead to cell death of *Leishmania* parasites and could explain the antileishmanial activity of the compounds **1** and **2** tested against *L*. *major*.

**Fig 6:**
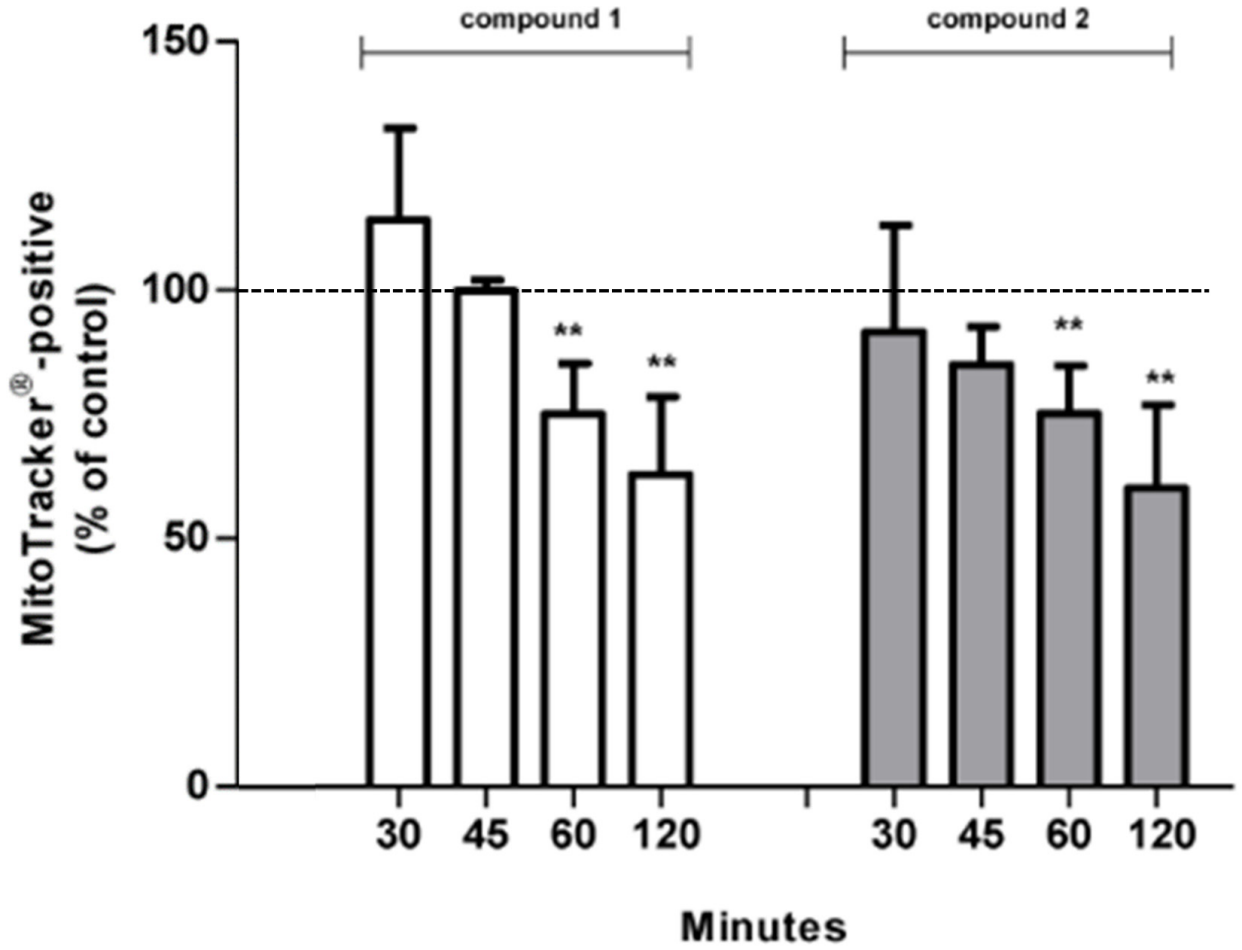
The tested compounds 1 and 2 affect mitochondrial integrity and membrane potential. Mitochondrial integrity of compound-treated *L*. *major* promastigotes was investigated using flowcytometric determination of MitoTracker Red CMXRos accumulation within active organelles. *L*. *major* promastigotes were incubated in 1% DMSO or treated with 100 μM compound 1 or 2 for indicated time points prior to MitoTracker Red CMXRos staining. MitoTracker-positive cell populations were determined by histograms analysis and were presented as percentage of control cells (set as 100%, inserted line).

For this reason, *L*. *major* promastigotes were treated either with DMSO or with compound **1** or **2** for 30 min, 45 min, 60 min, and 120 min prior to MitoTracker Red CMXRos fluorometric staining (Fig 6). As the accumulation of MitoTracker Red CMXRos indicates functional mitochondria, alterations of its function were detected over time in compound-treated *L*. *major* promastigotes. A significant drop in MitoTracker-positive cells has been observed in compound **1**-treated parasites compared to parasites treated with DMSO that were set as 100% functional mitochondria (Fig 6). The reduction of MitoTracker-positive cells after 60 min to 75% indicated alterations of mitochondrial functions that are becoming more prominent after 120 min of compound **1** treatment (63%).

Similar to these observations, also compound **2**-treatment altered the mitochondrial function of the parasite, as after 60 min and 120 min 75.2% and 60.2% MitoTracker-positive cells were observed, respectively (Fig 6). Differences between DMSO- and compound-treated promastigotes have been observed as early as 60 min. As MitoTracker Red CMXRos stains the mitochondria of live cells and its accumulation depends on the mitochondrial transmembrane potential it can be concluded that treatment with these compounds mediated a membrane depolarization in *L*. *major* promastigotes as a decreased accumulation of MitoTracker was observed.

These results support the hypothesis that the antileishmanial activities of the compounds tested in the present study are mitochondria-associated as a significant depolarization of the mitochondrial transmembrane potential was observed in compound-treated *L*. *major* promastigotes.

### Loss of cell membrane integrity in *L*. *major* promastigotes because of compound 1- and 2-treatment

The type of cell death induced by the compounds **1** and **2** was investigated using flow cytometric approaches. Since the translocation of PS to the outside of the cellular membrane without simultaneous loss of membrane integrity represents one of the features of early apoptosis in mammalian cells [28], staining with AV (binds to PS) and PI (DNA-binding) was applied to discriminate between four *Leishmania* populations as defined elsewhere [29]: live (AV^-^/PI^-^), necrotic/late apoptotic (AV^+^/PI^+^), necrotic (AV^-^/PI^+^) and early apoptotic cells (AV^+^/PI^-^) using per definition miltefosine-treated parasites as positive control for apoptosis induction in *Leishmania* [30].

**Fig 7:**
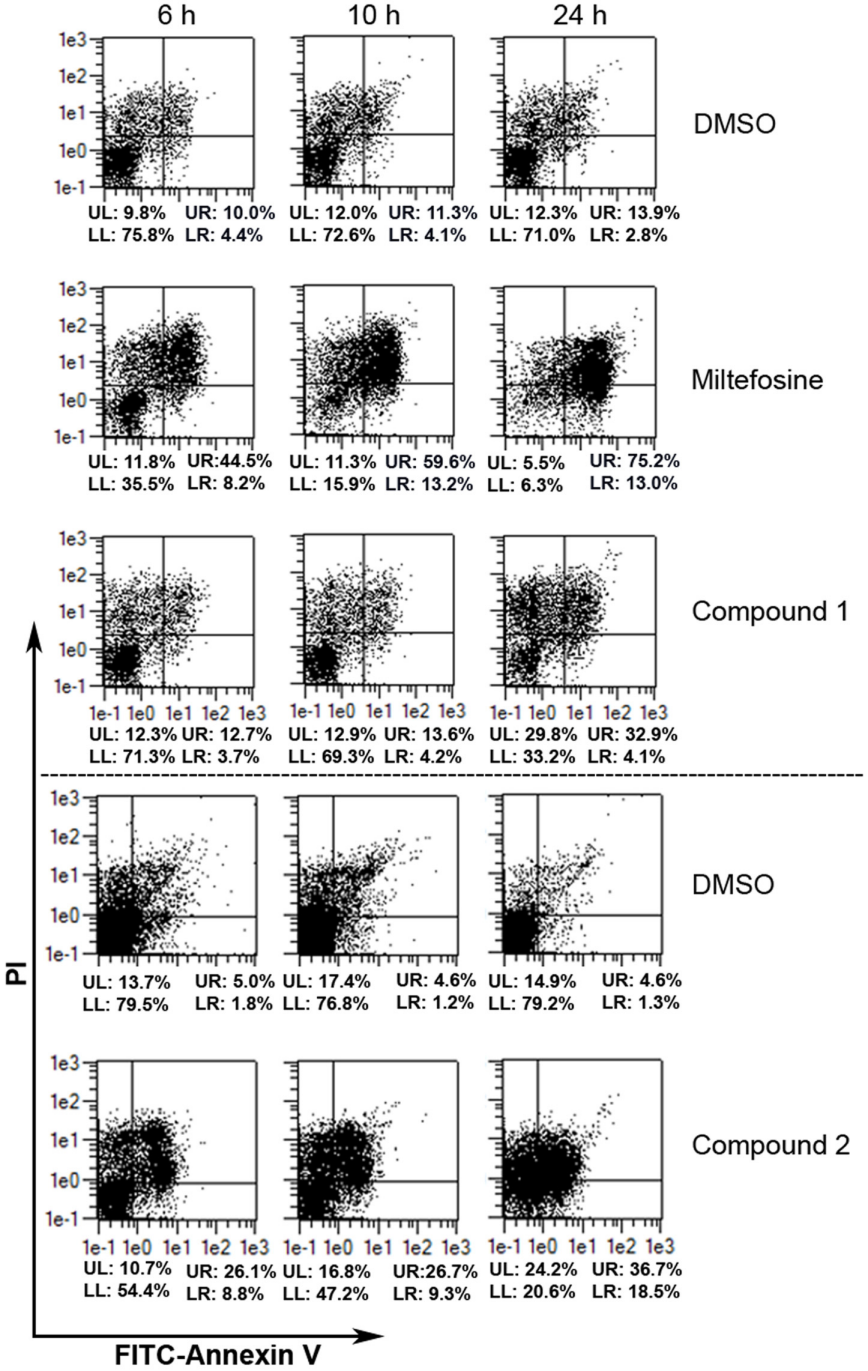
Compound-associated cell death is time-dependent. *L*. *major* promastigotes were treated with solvent control (1% DMSO), apoptosis inducer miltefosine (122.7 μM), compound 1 (100 μM), and 2 (100 μM) in a time course experiment for 6 h, 10 h, and 24 h. Cells were harvested, stained with AV and PI and subsequently analyzed by flow cytometry. Analysis for compound 2 was performed in an independent experiment. Percentage for each quadrant is written below the corresponding dot blot figure. Lower-left (LL): live cells (AV^-^/PI^-^); upper-left (UL): early necrotic cells (AV^-^/PI^+^); upper-right (UR): necrotic/late apoptotic cells (AV^+^/PI^+^); and lower-right (LR): early apoptotic cells (AV^+^/PI^-^).

After exposure of *L*. *major* promastigotes to bornyl cinnamate (**1**) and bornyl 3-phenylpropanoate (**2**) for 24 h, a percentage of only 33% and 21% cells were alive (AV^-^/PI^-^), respectively, whereas at the same time under normal growth conditions 71% and 79% of cells were unaffected und alive (Fig 7). Compound-mediated cell death was found to increase over time. Compound **1** treatment for 24 h resulted in increased cell permeability in 30% of all cells being characterized as necrotic (AV^-^/PI^+^) whereas additional binding of AV to the parasites surface was observed in 33% of all treated cells, leading to necrotic/late apoptotic (AV^+^/PI^+^) cells (Fig 7). These phenotypes increased over time, especially between the late time points from 10 h to 24 h of incubation. Interestingly, compound **2** induced a broad spectrum of phenotypes; after 24 h of treatment 24% of all cells showed per definition a necrotic (AV^-^/PI^+^), 19% per definition an early apoptotic (AV^+^/PI^-^), and 37% per definition a necrotic/late apoptotic cell death phenotype (AV^+^/PI^+^) (Fig 7). These phenotypic effects increased over time and were predominant after 24 h of incubation.

The presence of miltefosine induced a strong affinity of the parasitic surface towards AV in 88% of the *L*. *major* promastigotes within 24 h. Between 6 h and 24 h a substantial shift from 8% to 13% of early apoptotic cells (AV^+^/PI^-^) and a shift from 45% to 75% of necrotic/late apoptotic cells (AV^+^/PI^+^), respectively, was observed (Fig 7). Miltefosine is described as an inducer of early apoptosis in *L*. *major* using AV as read out [30]. Here, miltefosine induced a broad spectrum of cell deaths with a predominant late necrotic/late apoptotic phenotype (AV^+^/PI^+^).

In summary, investigations in *L*. *major* promastigotes treated with bornyl cinnamate (**1**) and bornyl 3-phenylpropanoate (**2**) for 24 h revealed their antileishmanial *in vitro* activity. Per definition, compound **1** induced a necrotic cell death phenotype and compound **2** a miltefosine-like cell death being per definition associated with elevated levels of early apoptotic cells. The mechanism underlying the antileishmanial activity of compound **1** and **2** is associated with mitochondria deformation and inhibition of its transmembrane potential with the consequence of cell death as defined by the loss of membrane integrity in *L*. *major* promastigotes.

## Discussion

Although leishmaniasis is one of the infectious diseases causing 20.000 to 30.000 deaths each year, it still belongs to the tropical neglected diseases [1]. Indeed, there is a huge clinical need, but no adequate treatment is available against leishmaniasis, mainly because the efficacy of the chemotherapy is limited by the continuous development of drug resistance to the first-line drugs [4]. Traditionally, different cultures around the world have been using native plants for treatment of systemic forms of leishmaniasis through oral administration of the crude extracts, while the cutaneous infections have been treated with topical preparations of the same. Therefore, the development of new antileishmanial therapies could involve the use of compounds present in plants of the endemic zones of leishmaniasis.

The majority of drug candidates with promising *in vitro* activity are either toxic or not efficient in experimental animal models. Those models are widely used to evaluate the antileishmanial efficacy of a new treatment with regard to the route of drug administration in correlation to the site of infection, and the treatment regimen. Among a pool of animal models, mostly mice are used for antileishmanial drug studies [31]. Some of the advantages of using inbred mice are the high reproducibility of the infection, the need of only few amounts of the tested drugs due to the small weight of the mice, and the contemporary evaluation of the drug efficacy within 2–5 weeks. *L*. *major* infection studies in BALB/c mice are most commonly chosen in drug discovery studies against experimental CL, as BALB/c mice are highly susceptible to the infection with no spontaneous recovery [32]. The cutaneous manifestation of a *L*. *major* infection is accompanied by footpad swelling at the site of infection. A reduction of swelling after drug administration is frequently associated with therapeutic elimination of parasites in the footpad and therefore used as first clinical readout for drug efficacy. However, foot pad swelling characterizes only indirectly the therapeutic effect. Theoretically, the reduction of inflammation can be uncoupled from the parasite burden in the infected footpad. Therefore, it is very important to determine the parasite burden directly within the infected foot pad and the inner organs of mice by LDA assay.

During this study we showed for the first time that phenylpropanoic acid bornyl ester (**2**) was efficient to treat *Leishmania* infections *in vivo*. Compound **2** tested against *L*. *major* in BALB/c mice was highly efficient, as the susceptible BALB/c mice showed a healing phenotype with reduced footpad swelling and parasite burdens. After a promising healing phenotype was observed in mice treated with compound **2** and the lack of apparent side effects *in vivo*, we further investigated the antileishmanial mechanism of action by *in vitro* approaches.

We first investigated cell morphological changes in promastigotes of *L*. *major* upon treatment with the compounds **1** and **2** and compared them with the effect induced by miltefosine. Presence of vacuole-like structures was evident for *L*. *major* promastigotes treated with the tested compounds and a massive cell death was visible after 24 h of incubation as compared to miltefosine-treated parasites.

Moreover, TEM revealed that the vacuole-like structures present in promastigotes of *L*. *major* after treatment with compound **1** and **2** corresponded to swollen mitochondria containing the kinetoplast DNA. Previous studies have shown that after treatment of *L*. *major* promastigotes and amastigotes with licochalcone A, a structurally similar constituent of roots and rhizomes of different plants, mitochondria of the cells were swollen as observed by electron microscopy [33]. Recent studies demonstrated that the effect of many antileishmanial drug candidates led to mitochondrial destabilization of the *Leishmania* parasite [34, 35]. These findings support the approach of the identification and evaluation of mitochondria-targeting drug candidates, as mitochondrial integrity and functionality is essential for *Leishmania* survival [36, 37].

The two compounds tested in the present study were found to induce cell death in *L*. *major* promastigotes through a combination of multiple features, such as shrinking of cells, mitochondrial swelling and loss of cell membrane integrity. Cell death is mainly defined by either the loss of cell membrane integrity as demonstrated by the incorporation of the vital dye PI or the degradation into distinct apoptotic bodies and vesicles [38]. The loss of membrane integrity has been shown by PI incorporation into compound **1-** and **2-**treated *L*. *major* promastigotes whereas apoptotic body formation within the parasites was not observed by TEM. Regulated cell death mechanism like apoptosis is described for mammalian cells based on the specificity of AV to its ligand PS [39], consequently serving as a specific marker for apoptosis. It is controversially discussed whether AV is a suitable marker for regulated cell death in *Leishmania* parasites [40, 41]. One reason for this concern is that PS was not yet identified in membrane fractions and lipid extracts, if at all in very low levels of *L*. *major* promastigotes [42]. Furthermore, it has been shown that PS is not the only ligand for AV, but also phospholipids like phosphatidylethanolamine and phosphatidylinositol are reported to be recognized by AV on the cell surface of *Leishmania* [42]. Additional investigations on molecular mechanisms could define appropriate *Leishmania*-specific readouts for regulated or unregulated cell death mechanisms [40]. Our flow cytometry analysis data suggested that compound **1**- and **2**- treated *Leishmania* promastigotes underwent apoptosis and necrosis, respectively. A similar result with both kinds of cell death was observed after treatment with miltefosine, which is a well-known apoptosis inducer in *Leishmania* parasite [30]. However, there is an ample discussion if an apoptotic cell death really exists in *Leishmania* parasites. Regarding this controversy it has been suggested by Proto and colleagues [40] to classify protozoan cell death as unregulated cell death as long as no molecular signaling mechanism proves regulated cell death processes. Taking these concerns into account, the antileishmanial activity of the compounds **1** and **2** leading to cell death is definitely based on severe mitochondrial alterations and the loss of the mitochondrial transmembran potential (ΔΨ_m_).

Beside target-based drug design, plant-derived compounds with antileishmanial activity have been described. Extracts from various plants like *Croton cajucara* [43, 44], *Zanthoxylum chiloperone* [45], *Chinese licorice* [46], and *Pera benensis* [47] have proven *in vitro* and *in vivo* activities against different strains of *Leishmania*. Apparently, the cytotoxicity of naturally derived compounds against host cells represents one obstacle of drug development [48]. *V*. *wallichii*-derived extracts possess a broad range of antimicrobial activity against e.g. *Staphylococcus aureus*, *Bacillus subtilis*, and *Escherichia coli* [49, 50]. Antiparasitic activity of *V*. *wallichii* chloroform root extracts was explored in studies against *Leishmania*, where significant antileishmanial activity against *L*. *donovani* and *L*. *major* promastigotes and intracellular *L*. *major* amastigotes was identified [10]. Leaf extracts from *Pluchea spp*., belonging to the class of *Asterids* like *V*. *wallichii*, inhibited the proliferation of *L*. *amazonensis in vitro* and the i.p. administration of 100 mg/kg/day is sufficient to prevent lesion development and to reduce parasite burden *in vivo* [51].

Furthermore, plants synthesize chalcones which are structurally related to the cinnamic acid derivatives tested in our study. Chalcones are precursors for flavonoids which are part of the biological defense mechanisms against plant pathogens [52]. Chalcones show antibacterial [53], antimalarial [54] and antileishmanial [55] activities among others. *In vitro*, the antileishmanial activity of chalcones isolated from *Chinese licorice* against *L*. *major* and *L*. *donovani* is related to mitochondrial alterations [56]. Synthetic analogues of chalcone isolates of *Crotalaria ramosissima* show enhanced *in vitro* and *in vivo* activity as compared to the natural chalcone [57]. Antileishmanial *in vivo* activity of chalcones is demonstrated against *L*. *donovani* infections in hamster and against the predominant causative agent of leishmaniasis in Latin America, *L*. *braziliensis* [55, 56]. Natural or synthesized compounds with structural similarities, like cinnamic acid derivatives and chalcones, show activity against a broad range of *Leishmania* parasites, indicating their potential as lead structures for the development of chemotherapeutic approaches against *Leishmania* infections. However, the structure of chalcones and cinnamic acid derivatives comprises a reactive Michael system, which is prone to unselective attacks by nucleophilic groups of proteins e.g. thiol groups of the amino acid cysteine or glutathione, thereby being able to give false-positive activity which is regarded as a PAINS problem [58].

To summarize, the process of the identification and evaluation of the antileishmanial compounds **1** and **2** started with the fractionation of crude root extracts from *V*. *wallichii*, a well-known plant used in traditional medicine. A semi-pure fraction of a chloroform extract was found to exhibit leishmanicidal *in vitro* activity against *L*. *major* and *L*. *donovani* [10]. Subsequent studies identified caffeic acid bornyl ester with antileishmanial *in vitro* activity [11]. SAR studies prompted the synthesis of less toxic but leishmanicidal compounds relying on the originally extracted structure from *V*. *wallichii* [12]. To eliminate possible unselective effects due to the reactive Michael system of cinnamic acid, compound **2** was synthesized without this moiety. Thus, cytotoxicity was reduced while antileishmanial activity was preserved. The successful evaluation of this compound in an experimental mouse model against *L*. *major* infections is an important step in the lead optimization and development. Preliminary studies in *L*. *donovani* infected BALB/c mice treated with the compounds **1** and **2** showed also promising antileishmanial *in vivo* activities, but need further evaluation. Primarily the hydrocinnamic acid derivative (**2**) shows promising *in vitro* activity while affecting and altering the mitochondria of *Leishmania*, making it a potent lead structure for the development of antileishmanial chemotherapeutic approaches.

## Supporting Information

S1 Checklist NC3Rs ARRIVE guidelines checklist. (PDF)
